# Intraocular Bleeding in Patients With Atrial Fibrillation Treated With NOACs VS. Warfarin: A Systematic Review and Meta-Analysis

**DOI:** 10.3389/fcvm.2022.813419

**Published:** 2022-06-01

**Authors:** Fuwei Liu, Yupei Zhang, Jun Luo, Yue Zhou

**Affiliations:** ^1^Department of Cardiology, The Affiliated Ganzhou Hospital of Nanchang University, Ganzhou, China; ^2^Second Clinical Medical College, Nanchang University, Nanchang, China; ^3^State Key Laboratory of Ophthalmology, Zhongshan Ophthalmic Center, Sun Yat-sen University, Guangzhou, China

**Keywords:** atrial fibrillation, non-vitamin K oral anticoagulants, warfarin, intraocular bleeding, meta-analysis

## Abstract

**Background:**

Intraocular bleeding is a devastating adverse event for patients with atrial fibrillation (AF) receiving anticoagulant therapy. It is unknown whether non-vitamin K oral anticoagulants (NOACs) compared with warfarin can reduce the risk of intraocular bleeding in patients with AF. Herein, we conducted a meta-analysis to evaluate the effect of NOACs vs. warfarin on intraocular bleeding in the AF population.

**Methods:**

Studies were systematically searched from the Embase, PubMed, and Cochrane databases until April 2022. We included studies if they enrolled patients with AF and compared the intraocular bleeding risk between NOACs and warfarin and if they were randomized controlled trials (RCTs) or observational cohort studies. The random-effects model was chosen to evaluate the pooled odds ratios (ORs) and 95% confidence intervals (CIs).

**Results:**

A total of 193,980 patients with AF from 5 randomized controlled trials (RCTs) and 1 cohort study were included. The incidence of intraocular bleeding among AF patients treated with warfarin and NOACs was 0.87% (*n* = 501/57346) and 0.61% (*n* = 836/136634), respectively. In the pooled analysis with the random-effects model, the use of NOACs was not significantly associated with the risk of intraocular bleeding (OR = 0.74; 95% CI 0.52–1.04, *P* = 0.08) compared with warfarin use. In addition, the sensitivity analysis with the fixed-effects model suggested that NOAC users had a lower incidence of intraocular bleeding than patients with warfarin (OR = 0.57; 95% CI 0.51–0.63, *P* < 0.00001).

**Conclusions:**

Our current meta-analysis suggested that the use of NOACs had no increase in the incidence of intraocular bleeding compared with warfarin use in patients with AF. Whether the use of NOACs is superior to warfarin needs more research to confirm.

## Introduction

Atrial fibrillation (AF) has become the most common arrhythmia that affects people worldwide. Patients who have been diagnosed with AF are more prone to suffer from thromboembolic events (1, 2). As such, we urgently need to find an appropriate treatment that can prevent the risk of thromboembolism in patients with AF (3, 4). Although warfarin, a kind of vitamin K antagonists, has already proven to be practical for thromboprophylaxis (5), it still has many disadvantages (e.g., interactions with other drugs or food, frequent monitoring of international normalized ratio) (6, 7). Recently, non-vitamin K oral anticoagulants (NOACs; i.e., dabigatran, rivaroxaban, apixaban, and edoxaban) have been widely used in patients with AF. Newly published guidelines consistently recommend the use of NOACs as the criterion for anticoagulant therapy in patients with AF in terms of their effectiveness and safety compared with warfarin (2, 3, 8).

Although NOACs are safer than warfarin for AF-related stroke prevention (9), the concern of bleeding risks still remains in the NOAC users. In patients with AF receiving anticoagulant therapy, a rare but serious complication of NOACs is intraocular bleeding (10, 11), which may cause visual acuity impairment and sometimes require surgical intervention if it deteriorates. Although three prior meta-analyses by Caldeira et al. (12), Sun et al. (13), and Phan et al. (14) have compared the risk of intraocular bleeding caused by NOACs and warfarin, they have yielded different results. Sun et al. (13) included 12 studies with a sample size of 102,627 patients with AF or venous thromboembolism (VTE) and found that NOACs could reduce the incidence of intraocular bleeding by up to 20% compared with warfarin. In contrast, Caldeira et al. (12) included 17 studies with 117,563 patients with AF or VTE, and claimed that there was no difference in intraocular bleeding between NOACs and warfarin. Phan et al. (14) conducted a network meta-analysis by including 102,617 patients with AF or VTE from 12 RCTs (11,746 treated with apixaban, 18,132 with edoxaban, 11,893 with rivaroxaban, 16,074 with dabigatran, 18,389 with switched NOACs and 44,764 with warfarin). They concluded that only edoxaban was associated with a significantly diminished risk of intraocular bleeding compared with warfarin. Moreover, a prospective cohort study conducted by Campello et al. (15), enrolling 275 cases and 322 controls, found that there was a slightly increased incidence of bleeding in thrombophilia patients treated with NOACs. Subsequently, a large observational cohort study by Park et al. (16) enrolled 27,496 patients with warfarin and 93,691 NOAC users and concluded that the risk of intraocular bleeding was lower in the NOAC group. It is still unclear whether the use of NOACs compared with warfarin can reduce the risk of intraocular bleeding in patients with AF. In the present meta-analysis, we re-evaluated the effect of NOACs vs. warfarin on intraocular bleeding in the AF population. Furthermore, we only included patients diagnosed with AF, rather than patients with AF or VTE. Not only RCTs but also observational cohort studies were included in our analysis.

## Methods

This meta-analysis and systematic review were performed according to the PRISMA (Preferred Reporting Items for Systematic Reviews and Meta-analyses) items (17). Since the results of studies included in this meta-analysis have been published, we did not need to provide ethical approval.

### Strategy of Literature Search

In order to find studies comparing the effects of NOACs and warfarin on patients with AF, we conducted a systematic search of articles published in the PubMed, Embase, and Cochrane databases before April 2022. The search terms were included as follows: 1) novel oral anticoagulants, non-vitamin K oral anticoagulants, direct oral anticoagulants, apixaban, edoxaban, dabigatran, rivaroxaban; 2) vitamin K antagonists, warfarin; and 3) atrial fibrillation. The detailed search strategies based on electronic databases were provided in Supplementary Table 1. There were no language restrictions in the search process.

### Eligibility Criteria

We included randomized controlled trials (RCTs) or observational cohort studies that reported the effect of NOACs (dabigatran, rivaroxaban, apixaban, or edoxaban) compared with warfarin in non-valvular AF patients. We chose studies that used intraocular bleeding as the outcome, which was defined as major bleeding. In this meta-analysis, only subretinal hemorrhage, vitreous hemorrhage, hyphema, and suprachoroidal hemorrhage were considered the major bleeding event, precluding minor uncomplicated bleedings (e.g., subconjunctival hemorrhages), which met the criteria set by the International Society on Thrombosis and Hemostasis (18). Studies focusing on AF patients with ablation, cardioversion, or left-atrial appendage were excluded. Certain publication types with insufficient data (e.g., comments, reviews, letters, case reports, expert opinions, and editorials) were also excluded.

### Data Extraction

After retrieving the literature, two reviewers screened them independently through title and abstract for the potential studies and then did a full-text reading to find the literature that met the requirements. Disagreements were resolved through discussion or consultation with a third researcher. Two reviewers extracted the following data: author, the year of publication, data source, study design, patient information, type and dosage of NOACs, follow-up period, number of events, and sample size.

### Quality Assessment

The bias risk of RCTs was assessed using the Cochrane Collaboration's tool on the selection bias, performance bias, detection bias, attrition bias, reporting bias, and other biases. For each domain of the Cochrane Collaboration's tool, the bias risk was scored as “low,” “unclear,” or “high” risk. For observational cohort studies, the Newcastle-Ottawa Scale (NOS) tool was used to assess the study quality. The NOS tool had a total of 9 points from 3 major sections: the selection of cohorts (0-4 points), the comparability of cohorts (0-2 points), and the assessment of the outcome (0–3 points). In this meta-analysis, the study with a NOS score of ≥ 6 points was defined as moderate to high quality, and a NOS score of < 6 points was considered a low quality (19).

### Data Analysis

The statistical heterogeneity across the included studies was assessed using the *P*-value in the Cochrane Q test and the I^2^ statistic. A *P*-value of <0.1 or I^2^ value of > 50% suggested significant heterogeneity. For each included study, we collected the number of events and the sample size in the warfarin- or NOAC- groups, which were pooled by the random-effect model in consideration of the substantial heterogeneity across the included studies. The pooled results were expressed as the odds ratios (ORs) and 95% confidence intervals (CIs). In the sensitivity analysis, we re-performed the above-mentioned analysis using a fixed-effects model. Moreover, we performed the sensitivity analysis by deleting data from a single study to analyze the impact of one single study on the combined effect size. According to the Cochrane handbook, the publication bias was not formally assessed when the number of the included studies was < 10.

All the statistical analyses were performed using the Review Manager version 5.4 software (the Cochrane Collaboration 2014, Nordic Cochrane Centre Copenhagen, Denmark; https://community.cochrane.org/). In this study, a *P*-value of < 0.05 indicated statistical significance.

## Results

### Study Selection

**Figure 1:**
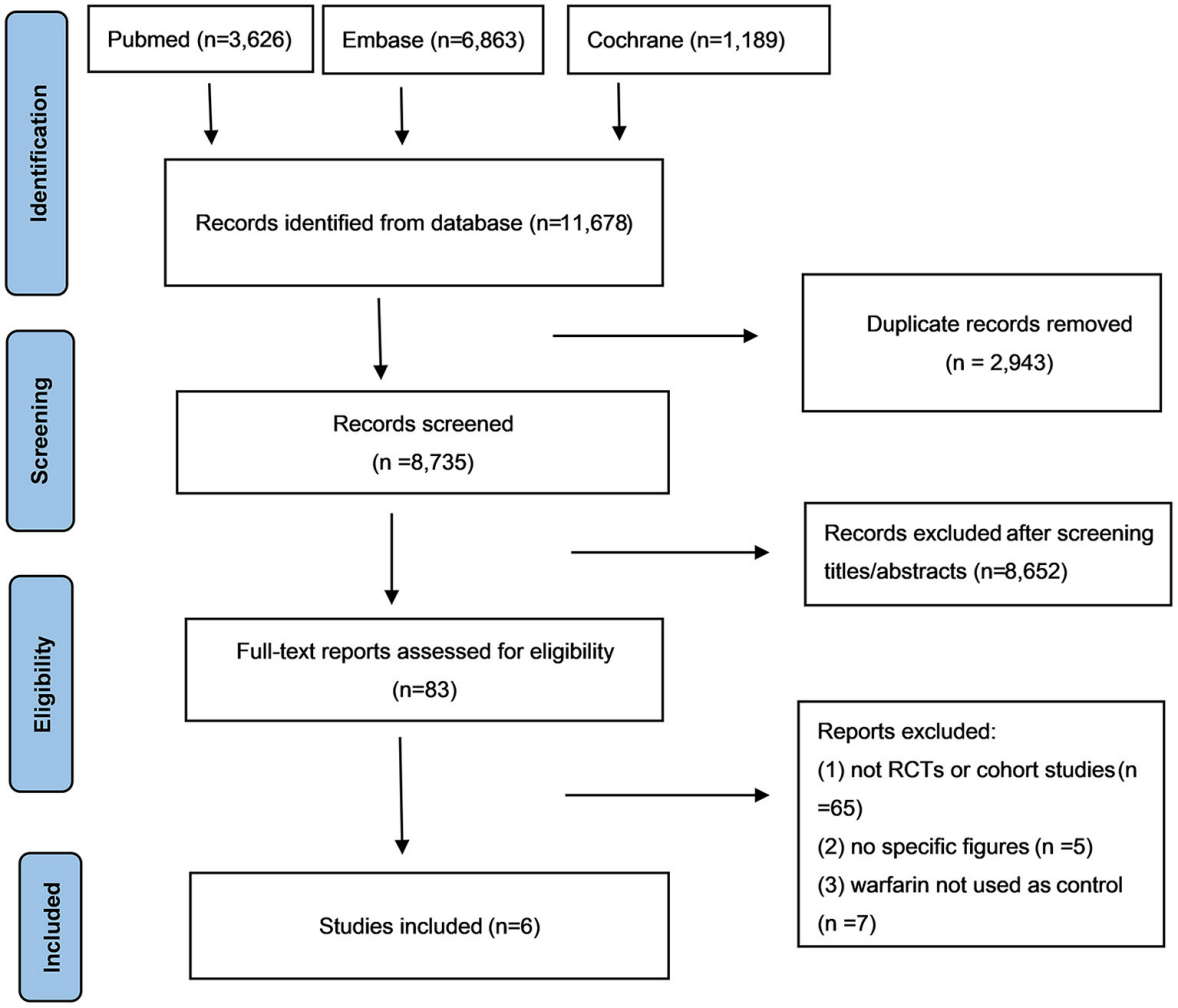
The process of literature search of this meta-analysis. NOACs, novel oral anticoagulants; CI, confidence interval.

After a careful literature search, a total of 11,678 articles were initially selected from the electronic database. Among them, 2,943 articles were excluded due to the repeated selection, and 8652 were eliminated after the screenings of the titles and abstracts. Subsequently, 77 articles were precluded after the full-text screenings because (1) studies were not RCTs or observational cohorts (*n* = 65); (2) no specific data were given (*n* = 5); (3) warfarin was not used as the reference (*n* = 7). Finally, a total of 6 studies [5 RCTs (20–24) and 1 observational cohort study (16)] were selected in this meta-analysis (Figure 1).

### Study Characteristics

The baseline characteristics of the included studies are listed in Table 1. The publication date of these articles ranged from 2009 to 2020, and the sample size ranged from 1,278 to 121,187. Specifically, Connolly et al. (20) included a total of 18,113 patients diagnosed with AF (6,022 patients on warfarin and 12,091 patients on NOACs). Granger et al. (21) enrolled 18,201 patients with AF, but 9,052 patients with warfarin and 9,088 patients with NOACs were included in this meta-analysis. Patel et al. (22) studied 14,264 patients with AF, including 7,111 patients treated with NOACs only and 7,125 patients treated with warfarin only. A total of 1,278 patients with AF were included in the study by Hori et al. (23), half of whom were treated with NOACs and half with warfarin. Giugliano et al. (24) studied 21,105 patients diagnosed with AF, including 7,012 patients who received warfarin and 14,014 patients who received NOACs. The study by Park et al. (16) included 121,187 patients with AF (27,496 on warfarin and 93,681 on NOACs). Among the various outcome, subretinal hemorrhage, vitreous hemorrhage, hyphema, and suprachoroidal hemorrhage were considered intraocular bleeding (18), precluding minor uncomplicated bleedings.

**Table 1:** The baseline characteristics of the selected studies.

**Included studies**	**Data source**	**Region**	**Study design**	**Type of patient**	**Oral anticoagulants**	**Average age**	**Male (%)**	**TTR (%)**	**No. of events**	**No. of patients**	**Follow-up time (y)**	**Study quality**
Connolly et al. (20)	RE-LY multicenter	NA(include44 countries)	RCT	AF	Warfarin	71.6	63.3	64	17	6022	2.0	Low risk
					Dabigatran	71.5	63.7	-	26	12091		
Granger et al. (21)	ARISTOTLE multicenter	NA	RCT	AF	Warfarin	70.0	65	62.2	19	9052	1.8	Low risk
					Apixaban	70.0	64.5	-	28	9088		
Patel et al. (22)	ROCKET AF multicenter	NA(include45 countries)s	RCT	AF	Warfarin	73.0	60.3	55	24	7125	1.9	Low risk
					Rivaroxaban	73.0	60.3	-	17	7111		
Hori et al. (23)	ROCKET AF multicenter	Japanese	RCT	AF	Warfarin	71.2	78.2	65	2	639	2.5	Low risk
					Rivaroxaban	71.0	82.9	-	3	639		
Giugliano et al. (24)	ENGAGE AF-TIMI 48 multicenter	NA	RCT	AF	Warfarin	72.0	62.5	64.9	37	7012	2.8	Low risk
					Edoxaban	72.0	61.7	-	46	14,014		
Park et al. (16)	the Health Insurance Review and Assessment service of Korea	Korea	Observational cohort study	AF	Warfarin	66.4	58.2	-	402	27496	2.7	NOS=7
					NOACs (apixaban, dabigatran, edoxaban, rivaroxaban)	72.5	52.7	-	716	93691	1.2	

*TTR, time in therapeutic range; RCT, randomized controlled trial; RE-LY, Randomized Evaluation of Long-Term Anticoagulation Therapy; ARISTOTLE, Apixaban for Reduction in Stroke and Other Thromboembolic Events in Atrial Fibrillation; ROCKET AF, Rivaroxaban Once Daily Oral Direct Factor Xa Inhibition Compared with Vitamin K Antagonism for Prevention of Stroke and Embolism Trial in Atrial Fibrillation; J-ROCKET AF, Japanese-Rivaroxaban Once Daily Oral Direct Factor Xa Inhibition Compared with Vitamin K Antagonism for Prevention of Stroke and Embolism Trial in Atrial Fibrillation; ENGAGE AF-TIMI 48, Effective Anticoagulation with Factor Xa Next Generation in Atrial Fibrillation–Thrombolysis in Myocardial Infarction 48;AF,Atrial Fibrillation*.

For the study quality assessment, all the 5 RCTs (20–24) had a low risk of bias, and the observational cohort by Park et al. (16) had an acceptable quality with a NOS of 7 points.

### Incidence of Intraocular Bleeding Between NOACs vs. Warfarin

**Figure 2:**
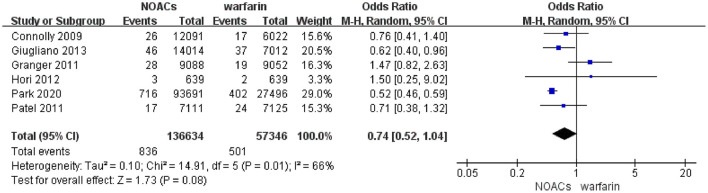
Forest plot of intraocular bleeding events in patients with AF with NOACs vs. warfarin using the random-effects model. NOACs, novel oral anticoagulants; CI, confidence interval.

In the pooled analysis, the incidence of intraocular bleeding in AF patients treated with warfarin and NOACs was 0.87% (*n* = 501/57,346) and 0.61% (*n* = 836/136,634), respectively. Our pooled results based on the random-effects model showed that the use of NOACs was not significantly associated with the risk of intraocular bleeding (OR = 0.74, 95% CI 0.52–1.04) compared with warfarin use (Figure 2). Of note, there was high heterogeneity across the selected studies (I^2^ = 66%).

After excluding the study by Granger et al., we found that the I^2^ value was reduced from 66% to 0%. The re-analysis after exclusion of the study by Granger et al. showed that NOACs distinctly diminished the rate of intraocular bleeding in patients with AF in comparison with warfarin (OR 0.54; 95% CI 0.48–0.61) (Supplementary Figure 1). In the sensitivity analysis with the fixed-effects model, the pooled results suggested that NOAC users had a lower incidence of intraocular bleeding compared with those patients with warfarin (OR = 0.57; 95% CI 0.51–0.63) (Supplementary Figure 2).

## Discussion

Our study was the first meta-analysis to investigate the effect of NOACs on the risk of intraocular bleeding in patients with AF compared with warfarin. According to our study based on the random-effects model, we found that there was no significant difference in the risk of intraocular bleeding in patients with AF between NOACs and warfarin. However, the pooled results changed in the sensitivity analysis when we used the fixed-effects model or excluded the study by Granger et al., and both suggested that NOAC users had a lower risk of intraocular bleeding than warfarin users.

In spite of the low incidence of intraocular bleeding in patients with AF after receiving anticoagulant therapy, it could lead to serious consequences once occurred. Massive intraocular bleeding was often associated with poor vision, and in some cases even required surgical intervention. Three previous predominant meta-analyses on this topic, conducted by Sun et al. (13), Caldeira et al. (12) and Phan et al. (14) respectively, carried out different conclusions. With an analysis involving 57,863 patients with AF or VTE from 12 studies, Sun et al. (13) found that novel oral anticoagulants could reduce the incidence of intraocular bleeding by up to 20% compared with warfarin, but Caldeira et al. (12) including 17 studies with a sample size of 117,563 patients with AF or TVE reported that NOACs couldn't diminish the incidence of intraocular bleeding in comparison with warfarin. However, although the risk estimates of Caldeira et al. cross unity, which indicated an absence of statistical significance, the wide 95% CIs and actual point estimates suggested that NOACs may still have a slight benefit (a decline by 16% in intraocular bleeding) compared with warfarin. Moreover, Phan et al. (14) conducted a network meta-analysis by including 102,617 patients with AF or VTE from 12 RCTs (11,746 of them were treated with apixaban, 18,132 with edoxaban, 11,893 with rivaroxaban, 16,074 with dabigatran, 18,389 with switched NOACs and 44,764 with warfarin). They concluded that edoxaban was associated with a significantly diminished risk of intraocular bleeding compared with warfarin, while Apixaban was the only NOAC associated with an increased risk of intraocular bleeding. Other NOACs were not different from warfarin. We believed that such a result may be caused by the small number of included studies, so we conducted an updated meta-analysis on this topic. In order to further investigate whether NOACs could reduce the incidence of intraocular bleeding in patients with AF compared with warfarin, we included data from newly published studies and then reperformed a meta-analysis on this topic. We did not include patients diagnosed with VTE because the effect of NOACs on patients with VTE and AF may be different, leading to the misestimation of the role of NOACs in the AF population. Unfortunately, it was shown that no significant difference was found between NOACs and warfarin in reducing the intraocular bleeding in patients with AF, according to the result of our meta-analysis.When in a sensitivity analysis, After excluding the study by Granger et al., we found that the I^2^ value was reduced from 66% to 0%. The re-analysis after exclusion of the study by Granger et al. showed that NOACs distinctly diminished the rate of intraocular bleeding in patients with AF in comparison with warfarin.It seems to suggest that NOACS is superior to warfarin, but due to the large heterogeneity across studies, we cannot draw this conclusion and more studies are needed to confirm.

Although our study did not directly demonstrate a benefit of NOACs, the actual point estimates and 95% CI of our results still suggested a potential benefit of NOACs in reducing the risk of intraocular bleeding. These results had clinical implications for ophthalmologists to correctly manage the patients receiving anticoagulant therapy, especially those with a high probability of intraocular bleeding. Unluckily, our work cannot answer the question of whether patients with AF treated with NOACs are at a lower risk of intraocular bleeding. This meta-analysis needs to be updated in the future with new research data. Current studies support that NOACs at least do not cause more harm than warfarin (25–27), and we predict that NOACs will become more popular among patients since there is no need to frequently draw blood to monitor the international normalized ratio. Nonetheless, clinicians still need to make sure appropriate doses of medication and always be aware of the possibility of bleeding symptoms.

Current studies did not explicitly explain the specific mechanisms of intraocular bleeding in patients receiving anticoagulation therapy. It has been speculated in the literature that NOACs only targeted one site in the coagulation, while warfarin targeted multiple sites (28). Due to the different mechanisms of action, it was also potentially suggested that the risk of intraocular bleeding may be different between NOACs and warfarin. In addition, the effects of different types (apixaban, edoxaban, dabigatran, rivaroxaban) (14, 29, 30) and doses (low, standard, high) (31–33) of NOACs may vary. Further detailed research is needed to confirm this hypothesis.

### Limitations of the Study

Overall, there are still some limitations in our study. First of all, no matter what kind of antithrombotic therapy was, intraocular bleeding was uncommon. As a result, the number of studies we included was limited and small. Similarly, the number of intraocular bleeding events in each trial was quite low. Second, the long-term effect of NOACs on intraocular bleeding could not be evaluated due to the short follow-up time of the included studies. Third, our study found no significant difference between NOACs and warfarin in influencing the risk of intraocular bleeding in patients with AF, but the actual point estimates and 95% CI suggested that NOACs still have a potential benefit. Fourth, data from both RCTs and observational studies were combined simultaneously, which may reduce the reliability of the results. Last but not the least, we did not differentiate between specific NOACs, which may have contributed to our lack of statistically significant results. After all, there are pharmacokinetic and pharmacodynamic differences among them, such as bioavailability, protein binding, and metabolism.

## Conclusions

Our current meta-analysis suggested that the use of NOACs had no increase in the incidence of intraocular bleeding compared with warfarin use in patients with AF. Whether the use of NOACs is superior to warfarin needs more research to confirm.

## Data Availability Statement

The original contributions presented in the study are included in the article/Supplementary Material, further inquiries can be directed to the corresponding author/s.

## Author Contributions

All authors listed have made a substantial, direct, and intellectual contribution to the work and approved it for publication.

## Conflict of Interest

The authors declare that the research was conducted in the absence of any commercial or financial relationships that could be construed as a potential conflict of interest.

## Publisher's Note

All claims expressed in this article are solely those of the authors and do not necessarily represent those of their affiliated organizations, or those of the publisher, the editors and the reviewers. Any product that may be evaluated in this article, or claim that may be made by its manufacturer, is not guaranteed or endorsed by the publisher.
