# Supplementary material for: Intraocular Bleeding in Patients With Atrial Fibrillation Treated With NOACs VS. Warfarin: A Systematic Review and Meta-Analysis
Source: Front Cardiovasc Med. 2022 Jun 1;9:813419. doi: 10.3389/fcvm.2022.813419 (PMC9199492; doi:10.3389/fcvm.2022.813419)
Supplement: Supplementary file 1 [file Data_Sheet_1.docx]

**Supplementary Table 1. The search strategies of this meta-analysis**

| **Databases** | **Queries** | **Number of studies** |
| --- | --- | --- |
| **PubMed** |  |  |
| #1 | "novel oral anticoagulants"[Title/Abstract] OR "non vitamin k oral anticoagulants"[Title/Abstract] OR "direct oral anticoagulants"[Title/Abstract] OR "apixaban"[Title/Abstract] OR "edoxaban"[Title/Abstract] OR "dabigatran"[Title/Abstract] OR "rivaroxaban"[Title/Abstract] | 14,296 |
| #2 | "vitamin k antagonists"[Title/Abstract] OR "warfarin"[Title/Abstract] | 30,177 |
| #3 | "atrial fibrillation"[Title/Abstract] | 83,681 |
| #4 | #1 and #2 and #3 | 3,626 |
| **Embase** |  |  |
| #1 | 'novel oral anticoagulants':ab,ti OR 'non-vitamin k oral anticoagulants':ab,ti OR 'direct oral anticoagulants':ab,ti OR 'apixaban':ab,ti OR 'edoxaban':ab,ti OR 'dabigatran':ab,ti OR 'rivaroxaban':ab,ti | 27,030 |
| #2 | 'vitamin k antagonists':ab,ti OR 'warfarin':ab,ti | 49,792 |
| #3 | 'atrial fibrillation':ab,ti | 146,067 |
| #4 | #1 and #2 and #3 | 6,863 |
| **Cochrane** |  |  |
| #1 | (novel oral anticoagulants):ti,ab,kw OR (non-vitamin k oral anticoagulants):ti,ab,kw OR (direct oral anticoagulants):ti,ab,kw OR (apixaban):ti,ab,kw OR (edoxaban):ti,ab,kw OR (edoxaban):ti,ab,kw OR (rivaroxaban):ti,ab,kw | 4,320 |
| #2 | (vitamin k antagonists):ti,ab,kw OR (warfarin):ti,ab,kw | 5,591 |
| #3 | (atrial fibrillation):ti,ab,kw | 14,065 |
| #4 | #1 and #2 and #3 | 1,189 |





**Supplementary figure 1**. **Forest plot of intraocular bleeding events in patients with AF with NOACs versus warfarin after excluding the study by Granger et al.**

**Abbreviations:NOACs=novel oral anticoagulants;CI=confidence interval;**

**
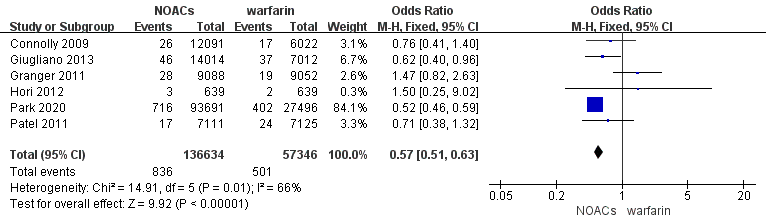
Supplementary figure 2. Forest plot of intraocular bleeding events in patients with AF with NOACs versus warfarin using the fixed-effects model**

**Abbreviations:NOACs=novel oral anticoagulants;CI=confidence interval;**
